# Functional Analysis of an Essential GSP1/Ran Ortholog Gene, *CpRan1*, from the Chestnut Blight Fungus *Cryphonectria parasitica* Using a Heterokaryon

**DOI:** 10.3390/jof7050332

**Published:** 2021-04-25

**Authors:** Yo-Han Ko, Jeesun Chun, Dae-Hyuk Kim

**Affiliations:** Department of Molecular Biology, Institute for Molecular Biology and Genetics, Jeonbuk National University, 567 Baekje-daero, Jeonju, Chonbuk 54896, Korea; kocineda@naver.com (Y.-H.K.); brainyjsc@gmail.com (J.C.)

**Keywords:** *Cryphonectria parasitica*, Ran, heterokaryon, hypovirulence

## Abstract

Functional analysis of a GSP1/Ran ortholog, *CpRan1*, from *Cryphonectria parasitica* was conducted. Genotype analysis revealed that the putative *CpRan1*-null mutant was a heterokaryotic transformant harboring two different types of nuclei, one with the wild-type *CpRan1* allele and the other with the *CpRan1*-null mutant allele. The mycelial growth and colony morphology of the heterokaryotic transformant was normal. Microscopic analysis of the resulting conidia (aseptate and monokaryotic asexual spores) demonstrated that although normal germinating spores were observed from conidia harboring a nucleus with the wild-type *CpRan1* allele, a number of residual conidia that did not germinate existed. Complementation analysis using protoplasts from the heterokaryon with the wild-type *CpRan1* allele confirmed that the *CpRan1* gene is essential to *C. parasitica*. Complementation analysis using the various *CpRan1* chimera constructs allowed us to perform a functional analysis of essential amino acids of the *CpRan1*. Among the four suggested essential amino acids, Lys-97 for ubiquitination was determined to not be an essential residue. Moreover, the *CpRan1*-null mutant allele was successfully complemented with mouse Ran gene, which suggested that the biological function of Ran gene is evolutionary conserved and that our heterokaryon rescue can be applied for the functional analysis of heterologous genes.

## 1. Introduction

*Cryphonectria parasitica* (Murrill) Barr, causes chestnut blight and has devastated chestnut forests and orchards in North America since the early 20th century [1]. However, *C. parasitica*, which contains a cytoplasmic single stranded RNA (ssRNA) virus, Cryphonectria hypovirus 1 (CHV1), has had lowered virulence, a phenomenon referred to as “hypovirulence” [2,3,4]. CHV1-infected *C. parasitica* displays diverse hypovirulence-associated symptoms, such as reduced pigmentation, sporulation, laccase production, and oxalate accumulation [5,6,7]. More interestingly, CHV1 can be transferred from the virus-containing hypovirulent strain to virus-free strain during hyphal fusion resulting in hypovirulence and its associated symptoms, which is an effective good model for naturally occurring biological control by mycovirus, also known as virocontrol [8,9].

Molecular analysis of *C. parasitica*–hypovirus interactions allowed us to ascribe the development of these viral symptoms to aberrant expressions of specific genes in the hypovirulent strain [10,11,12,13,14,15]. Due to the efficient genetic manipulations of *C. parasitica*, the availability of a high-quality *C. parasitica* genome sequence (http://genome.jgi-psf.org/Crypa2/Crypa2.home.html, accessed on 16 November 2016), and the application of an infectious cDNA copy of the hypovirus, *C. parasitica*–hypovirus interactions have been considered to be an ideal model to investigate fungus-virus interactions [16,17,18,19,20].

The Ran (Ras-related nuclear) protein is a highly conserved small GTP-binding nuclear protein and a vital component of the nucleocytoplasmic transport machinery that moves into and out of the cell nucleus during interphase and mitosis. In turn, Ran is known to play an important role in numerous cellular processes including various mitotic processes as well as antiviral immunity [21,22,23,24,25]. Ran primarily localizes in the nucleus and only a minor proportion is cytosolic in interphase cells, translocating between the cytosol and the nucleus through nuclear pore complexes. The concentration of RanGTP between the nucleus and cytosol is important for the directionality of nucleocytoplasmic trafficking. Nucleotide binding to Ran is tightly regulated by modulators such as Ran-binding proteins (RanBPs), guanine nucleotide exchange factor (RanGEF), and RanGTPase-activating protein (RanGAP). The strict compartmentalization of these modulating proteins allows them to maintain a sharp gradient in the concentration of RanGTP between the nucleus and the cytosol [26,27]. Recently, we have described how the gene encoding Ran-binding protein of *C. parasitica*, *CpRbp1*, was affected by CHV1 infection or supplementation with tannic acid, which is known to be abundant in the bark of chestnut trees and is defined as being one of the major barriers against pathogen infection [20]. Our functional analysis proved that *CpRbp1* is essential to biological processes [28].

Heterokaryosis, defined as the presence of two or even more genetically different nuclei in a common cytoplasm, is a unique genetic feature of fungi. A heterokaryon refers to a fungus that exhibits heterokaryosis by maintaining different types of nuclei in a cell. Heterokaryon is a useful genetic resource for nonsexual genetic variation. Although the ratio of nuclear genotypes in heterokaryons varies with a wide range and can be influenced by environmental conditions, a heterokaryon is effective in maintaining and even proliferating nuclei that contain a lethal genotype. In addition, heterokaryons can break down during the production of uninucleate spores, each of which can be cultured as a monokaryotic progeny. The maintaining, proliferating, and breaking down of different genotypes is specifically appropriate for the analysis of mutant nuclei in which a gene of interest is severely detrimental or lethal. These genetic processes provide the means of functional analysis via complementation with various chimeric constructs of the corresponding gene [28].

Regarding heterokaryon analysis, *C. parasitica* has the following advantages over other fungi: first, analysis of the natural population of *C. parasitica* reveals the presence of stable heterokaryons [29]; second, forced heterokaryon formation with mutant nuclei of an essential gene is successful during genetic manipulation [28]; third, the asexual spore of *C. parasitica* is a uninucleate single cell [30,31]. *C. parasitica* has a relatively high tendency to form heterokaryons, stably propagate them, and then easily break down each nuclei. These characteristics make this fungal system ideal for performing the functional analysis of morphogenic as well as essential genes.

Our previous pathoproteomic studies demonstrated that not all, but specific components of the Ran complex, such as RanBP1 and Ran, were affected by CHV1 infection and/or tannic acid supplementation. Although our recent study on RanBP revealed that the *CpRbp1* encoding RanBP is essential, no studies on the other components of the Ran complex have been conducted. Thus, we investigated the biological function of the Ran gene, which is also affected by CHV1 infection and tannic acid supplementation [20]. In this study, we describe heterokaryon formation with Ran gene mutant nuclei. We also detail our functional analysis of the Ran gene via complementation of the mutant nucleus with various Ran gene structures.

## 2. Materials and Methods

### 2.1. Fungal Strains and Growth

Strains of virus-free *C. parasitica* EP155/2 (ATCC 38755) and its genetically identical (isogenic) CHV1-713-containing hypovirulent UEP1 were maintained on a potato dextrose agar supplemented with L-methionine (0.1 mg/mL) and biotin (0.1 μg/mL) (PDAmb) at 25 °C with constant low light [18]. Endothia Parasitica (EP) complete medium was used for standard liquid culture [32]. The methods to prepare the primary inoculum for liquid cultures and culture conditions have been previously described [18]. Tannic acid induction of fungal strains was performed according to procedures previously described [20]. Briefly, mycelia cultured for 10 days on cellophane that had been layered on the top of PDAmb medium were transferred to a plate supplemented with an appropriate concentration of tannic acid [33]. Consistent with previous procedures, the mycelium was collected and lyophilized until it was used [34].

### 2.2. Quantitative Analysis of Transcript Accumulation Using Real-Time RT-PCR

To examine *CpRan1* gene expression levels, quantitative real-time PCR using reverse transcriptase (qRT-PCR) was performed using a GeneAmp 7500 sequence detection system (Applied Biosystems, Foster City, CA, USA) and a SYBR green mixture RT kit (Applied Biosystems, Foster City, CA, USA) as previously described [28]. Glyceraldehyde-3-phosphate dehydrogenase gene (*Gpd*) was used as an internal control [4,10,28,30,31]. Primer pairs for *Gpd* and *CpRan1* genes were indicated as RT-Gpd-F (forward) and RT-Gpd-R (reverse), as well as RT-Ran1-F1 (forward) and RT-Ran1-R1 (reverse), respectively (Supplementary Table S1). In the sample, transcript abundance, relative to the amount of *Gpd* gene, was calculated using the comparative threshold cycle method as previously described [35]. Analyses were conducted from three independent RNA preparations, in triplicate for each transcript, with primers specific for the *Gpd* and *CpRan1* genes.

### 2.3. Statistical Analysis

The accumulation of *CpRan1* transcripts of each strain were compared with hypovirus infection or tannic acid supplementation, analyzed by ANOVA using SPSS software (version 23.0, SPSS Inc., Chicago, IL, USA), and then comparison significance was determined using the Tukey honest significant difference (HSD) test at *p* < 0.01.

### 2.4. Cloning and Characterization of a GSP1/Ran Like Gene, CpRan1

Tandem mass analysis revealed the amino acid sequence of a selected protein spot [20]. The genome data base of *C. parasitica* (http://genome.jgi-psf.org/Crypa2/Crypa2.home.html, accessed on 16 November 2016) was analyzed to identify the gene encoding the determined amino acid sequence. In this study, we further investigated one of these spots corresponding to the *Saccharomyces cerevisiae GSP1.* We performed PCR amplification of this gene using primers CpRan1-gF1 (forward) and CpRan1-gR1 (reverse) (Supplementary Table S1). The resulting 5000-bp PCR amplicon was cloned into the pGEM-T Easy vector and sequenced.

To obtain the cDNA clone, PCR using reverse transcriptase (RT-PCR) was performed with primers CpRan1-cF1 (forward) and CpRan1-cR1 (reverse) (Supplementary Table S1). The resulting 651-bp cDNA amplicon was cloned and sequenced.

### 2.5. Southern and Northern Blot Analysis

Genomic DNA extraction and Southern blot analysis, implemented with the restriction enzyme *Eco*RV and a radioactive probe, were conducted as described in an earlier study [16].

RNA extraction from liquid cultures was conducted as previously described [36]. RNA extraction from mycelia mats grown on cellophane layered on top of appropriate solid media was performed as previously described [20]. The level of *CpRan1* transcript was normalized to that of glyceraldehyde-3-phosphate dehydrogenase (*Gpd*) of *C. parasitica* as an internal control for gene expression [37].

### 2.6. Target Gene Replacement Vectors and Fungal Transformation

Gene replacement using split-marker deletion cassettes was applied to study the biological function of the *CpRan1* gene [38]. Two molecular DNA cassettes, each of which contained a part of the hygromycin B phosphotransferase gene cassette (*hph*) fused the either the 2643-bp 5′- or 2576-bp 3′- flanking regions of the *CpRan1* gene, were prepared by overlap PCR [38] as follows: a 1055-bp PCR amplicon containing the 1035-bp 5′ flanking region of *CpRan1* was amplified using gene-specific primers Ran 5′-F1 and Ran 5′-R1 (Supplementary Table S1). A 1627-bp part of a selection marker gene containing the promoter and part of the N-terminus was amplified using primers Ran-Hph-F1 and Hph-R1 (Supplementary Table S1). The fusion of these two PCR amplicons was conducted using overlap extension PCR with primers Ran1-F1 and Hph-R1. A 2576-bp PCR fragment of the 980-bp 3′ flanking region and a 1596-bp part of the selection marker gene containing the terminator and part of the C-terminus were amplified and fused using primers Hph-F2/Hph-Ran-R2, Ran 3′-F2/Ran 3′-R2, and Hph-F2/Ran 3′-R2 (Supplementary Table S1). The resulting molecular cassettes were then used simultaneously to transform protoplasts of the *C. parasitica* EP155/2 strain.

Functional complementation of the *CpRan1*-null mutant using the wild-type allele was performed. The complementing vector pCRan was constructed by insertion of a 4615-bp *Not*I fragment of pSilent-Dual1G (pSD1G), which contained the geneticin resistance cassette [39], into *Not*I-digested pRan carrying a 5035-bp fragment with the full-length *CpRan1* gene. The resulting vector was then used to transform the putative *CpRan1*-null mutant.

Protoplast preparation and transformation were performed as previously described [16,18]. Transformants were selected from agar plates that were supplemented with 150 μg/mL hygromycin B (Calbiochem, San Diego, CA, USA) or 150 μg/mL geneticin (Invitrogen, Carlsbad, CA, USA), subcultured three to four times on appropriate selective media, and single spores were isolated when possible, as previously described [28,30]. PCR and Southern blot analysis of the transformants were conducted to confirm replacement and in-trans complementation of the *CpRan1* gene.

### 2.7. Staining and Microscopy

DAPI staining was performed as described previously [31,40]. Before imaging, adherent mycelia were fixed on coverslips with formaldehyde and washed briefly with distilled water. Mycelial staining was performed for 10 min at room temperature using 100 ng/mL 4,6-diamidino-2-phenylindole (DAPI, Sigma–Aldrich, St. Louis, MO, USA) and was followed by washing with distilled water. The slides were then mounted in phosphate buffered saline (PBS) containing 50% glycerol and 0.1% n-propyl-gallate and observed under a fluorescence microscope using a model LSM 880 in the Center for University-wide Research Facilities (CURF) at Jeonbuk National University (Carl Zeiss, Germany).

### 2.8. Ratio of Different Types of Nuclei

To estimate the ratio of different types of nuclei in heterokaryons, the putative heterokaryotic transformant was transferred every 5 days over 10 times on non-selective PDAmb and hygromycin B-containing selective PDAmb media. Each mycelium was grown on cellophane overlaid on the top of the appropriate media and harvested to extract genomic DNA. The nuclear ratio was confirmed through Southern blot analysis. The density of each band was quantified using Image J software (provided by the National Institutes of Health). In order to obtain conidia, successively cultured strains were transferred onto PDAmb and incubated for 2 weeks. Then, 100 conidia (as determined by a hemocytometer) were spread on PDAmb plates supplemented with or without hygromycin B. The resulting colony-forming units (CFUs) were counted to determine the number of wild-type nuclei. Three representative plates were selected for each transfer and analyzed statistically.

### 2.9. Construction of Complementary Vectors

Complementation assays of the *CpRan1*-null mutant were performed using genomic DNA, cDNA of *CpRan1*, and Ran cDNA of the mouse *Mus musculus*. The complementing vector based on genomic DNA, pCRan-gDNA, was constructed by insertion of a 5615-bp *Not*I fragment of pBluescript SK(-) containing the geneticin resistance cassette [39] into *Not*I-digested pGRan carrying a 5035-bp fragment with the full-length *CpRan1* gene. The complementing vectors based on cloned cDNA of *CpRan1* were constructed by insertion of a 1425-bp *Not*I-digested cDNA expression cassette consisting of a strong constitutive cryparin promoter (p188), 651-bp cDNA, along with a *trpC* terminator into a 4615-bp *Not*I fragment of the geneticin resistance cassette. In order to analyze the role of the selected amino acid residues in the protein product of the *CpRan1* gene, we used the Saccharomyces Genome Database (SGD; https://www.yeastgenome.org/locus/S000004284/protein, accessed on 14 May 2018) to select amino acid residues with post-translational modifications. Among seven suggested residues, we randomly selected four involved in succinylation (K25), ubiquitination (K101), ubiquitination (K125), and phosphorylation (S155) for analysis. In silico analysis using pair-wise comparison of amino acid sequences from GSP1 and CpRan1 genes revealed corresponding conserved amino acid residues for succinylation (at K21), ubiquitination (at K97), ubiquitination (at K121), and phosphorylation (at S151) within the protein product of the *CpRan1* gene. Four different chimeric structures harboring a point mutation at an appropriate amino acid residue were obtained by PCR amplification using a primer set that contained the corresponding mutated sequence. The complementing vector for the cDNA of mouse Ran was also constructed as that of the cDNA of *C. parasitica*. The resulting vectors were then used to transform the putative *CpRan1*-null mutant.

## 3. Results

### 3.1. Characterization of a Tannic Acid-Responsive and Hypoviral-Regulated CpRan1 Gene

Proteomic analysis of *C. parasitica* revealed a protein spot, which increased due to the hypovirus infection or tannic acid supplementation, and was identified as a Ran protein (GSP1/Ran) ortholog [20]. Inspection of the *C. parasitica* genome database (http://genome.jgi-psf.org/Crypa2/Crypa2.home.html, accessed on 16 November 2016) allowed us to determine the corresponding nucleotide sequence that encode the Ran protein ortholog. PCR amplification of a 5000 bp fragment, which was expected to contain the full-length *Ran* gene, followed by cloning and sequencing confirmed the nucleotide sequence. A near full-length cDNA was amplified using reverse transcription polymerase chain reaction (RT-PCR) with the primer pair CpRan1-cF1 and CpRan1-cR1 at 1 to 18 and 1031 to 1048. The resulting 656 bp amplicon was cloned and sequenced. Sequence comparison of the cDNA clone with the corresponding genomic clone revealed that the cDNA clone consisted of five exons, with four intervening sequences of 64 to 241, 335 to 396, 551 to 622, and 829 to 908 (relative to the start codon). These were equal to the computer prediction. The sequence around the first ATG was in good agreement with Kozak’s consensus sequence in that the nt -3 position was the A in CAACGATCATG. The putative poly (A) signal of AAATA was observed at 365 nt downstream of the stop codon.

The cloned gene has an open reading frame (ORF) of 651 bp that encodes 217 amino acid residues with an estimated molecular mass of 24.5 kDa and a pI of 6.11 (GenBank No. KAF3769082.1). Homology searches using the deduced amino acid sequence indicated that the protein product of the cloned gene is related to other fungal Ran homologs from *Trichoderma virens* (XP_013951777.1, 94.91% AA identity), *Fusarium beomiforme* (KAF4331629.1, 94.44%), *Colletotrichum asianum* (KAF0317623.1, 93.95%), *Verruconis gallopava* (XP_016218807.1, 92.89%), and *Aspergillus nidulans* (AAR08135.1, 91.04%). The multiple alignment of five closely related Ran proteins showing an E value ≈ 0.0 and Ran genes from *S. cerevisiae* revealed that the protein product of the cloned gene had a characteristic multi-domain structure, which consisted of four guanine nucleotide-binding domains, an effector domain, and an acidic C-terminal domain (Supplementary Figure S1A). The phylogram was supported with high bootstrap values suggesting a genuine evolutionary relationship (Supplementary Figure S1B). Together with the presence of conserved domains and a significant homology to known fungal Rans, the cloned gene was referred to as *CpRan1* for the *C. parasitica* Ran gene. In addition, inspection of the *C. parasitica* genome database revealed that no other gene contained the characteristic multi-domains and no gene except *CpRan1* showed high similarity to other known Ran proteins, which strongly indicated that the *CpRan1* gene is the only gene encoding the Ran protein.

### 3.2. Expression of CpRan1

**Figure 1:**
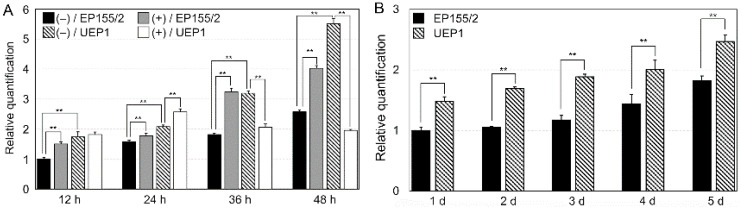
Expression analysis of *CpRan1*. (**A**) qRT-PCR analysis of the expression levels of *CpRan1* in response to CHV1 infection and TA supplementation. Changes in expression of *CpRan1* between the wild-type (EP155/2; indicated as solid bars) and CHV1-infected hypovirulent (UEP1; indicated as slashed bars) strains relative to levels of glyceraldehyde-3-phosphate dehydrogenase (*gpd*) are indicated; (+) and (−) indicate with and without TA supplementation, respectively. (**B**) qRT-PCR analysis of the expression levels of *CpRan1* during cultivation in standard liquid EP complete medium. Numbers at the bottom indicate hours after the transfer (**A**) and days in liquid culture (**B**). ** indicates a significant change at *p* < 0.01. Error bars indicate standard deviation based on three independent measurements.

Since our previous proteomic study showed down-regulation of the protein product of the *CpRan1* gene by either hypovirus infection or tannic acid supplementation [20], the accumulation of the *CpRan1* transcript of the wild-type EP155/2 strain and its isogenic CHV1-infected hypovirulent strain UEP1 were examined under corresponding conditions using qRT-PCR and Northern blot analysis (Figure 1A and Supplementary Figure S2). The qRT-PCR results indicated that a slight but significant increase in *CpRan1* transcript levels occurred in EP155/2 up to 24 h after the TA-supplementation. EP155/2 induction levels greatly increased at 36 h and 48 h after TA-supplementation. The accumulation of *CpRan1* transcripts increased even more significantly in the CHV1-infected UEP1 strain, which is isogenic to the wild type. No further induction, however, was observed in CHV1-infected UEP1 cultured on TA-supplemented media. Northern blot analysis revealed that the accumulation of *CpRan1* transcripts of the wild-type EP155/2 was increased 24 h after the TA supplementation. *CpRan1* transcript increases were also noted in UEP1, a slight rise being observed in UEP1 with the TA supplementation (Supplementary Figure S2). These results are consistent with those obtained through the qRT-PCR. Interestingly, a significant reduction in the accumulation of *CpRan1* transcripts was observed after 36 h in CHV1-infected UEP1 cultured on TA-supplemented media. These results indicate that hypovirus infection or TA supplementation alone upregulated the accumulation of the *CpRan1* transcript in *C. parasitica*. Their effect, however, on *CpRan1* expression was counteracted in combination. These results suggest that the expression of the *CpRan1* gene in planta is greatly affected by the CHV1 infection compared to its expression with the virus-free EP155/2. This interaction suggests that the expression of the *CpRan1* gene is important for fungal virulence.

We also examined the accumulation of transcripts of the *CpRan1* gene of the wild-type EP155/2 and CHV1-infected UEP1 strains under standard liquid-culture conditions (Figure 1B). The accumulation of the *CpRan1* transcript gradually increased in both EP155/2 and UEP1 strains as the culture proceeded. Significant upregulation of the *CpRan1* transcript was observed in the CHV1-infected UEP1 strain.

### 3.3. Screening and Identification of a CpRan1-Null Mutant

**Figure 2:**
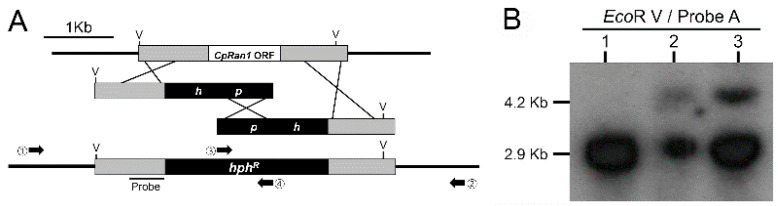
Restriction map and Southern blot analysis of the wild-type EP155/2 and the *CpRan1*-null mutant. (**A**) Restriction map of the *CpRan1* genomic region and the gene replacement split makers replacing the *CpRan1* ORF region with the hygromycin B resistance cassette flanked by 1035-bp and 980-bp fragments as the 5′- and 3′-flanking regions, respectively. The *CpRan1*-null mutant with the desired replacement at *CpRan1* is represented in the map with the expected changes in the size of the restriction fragments. Flanking regions and expected replacing ORF region are indicated by shadows and open boxes, respectively. *hph*^R^, indicated by the dashed box, represents the hygromycin B resistance cassette. Genes outside the replacement vector are indicated by lines. V represents restriction endonuclease *Eco*RV. (**B**) Southern blot analysis of the wild-type EP155/2 strain (lane 1) and two heterokaryotic *CpRan1*-null mutants (lanes 2, 3; note that in order to compare the intensity of hybridizing bands, two different amounts (10 μg and 20 μg) of genomic DNA were used for lanes 2 and 3, respectively.). Enzyme/probe combination is indicated above the line, and the probe A is indicated in the restriction map in the upper panel (**A**).

Understanding the biological function of the *CpRan1* gene was initiated by a comparison of the wild type with the *CpRan1*-null mutant, which was constructed by site-directed recombination during integrative transformation. A total of 142 putative transformants were obtained from agar plates that were overlaid with 150 μg/mL hygromycin B and passaged four times on selective media containing 50 μg/mL hygromycin B by successively transferring agar plugs containing actively growing young hyphae. All actively growing stable transformants were selected for further analysis. A total of 130 stable transformants were further screened by PCR using two pairs of primer sets designed for the detection of the mutant allele of the *CpRan1* gene, i.e., outer gene-specific and inner *hph* primers (Primers 1 and 4 and 3 and 2 in Figure 2A) corresponding to −1988 to −1967, 1222 to 1241, 446 to 465, and 2991 to 3012 (relative to the start codon of the *CpRan1* and *hph* genes). With one exception, all transformants showed the absence of PCR amplicons. The exception showed PCR amplicons of the expected sizes of 3608 bp and 3561 bp corresponding to the disrupted alleles of the *CpRan1* gene (Supplementary Figure S3A,B). When the putative *CpRan1*-null mutant was further examined by Southern blot analysis, however, two hybridizing bands were observed. One of these bands was the same size as the wild-type *CpRan1* allele, while the other band was the size of the expected *CpRan1*-null allele (Figure 2B). In addition, PCR analysis designed for the amplification of the replaced fragment by using outer gene-specific primers (Primers 1 and 2 in Figure 2A) revealed not one, but two bands presumably representing the wild-type allele and the mutant allele (Supplementary Figure S3C). Subsequent cloning and sequencing of these two PCR amplicons confirmed that the 5000 bp amplicon was the wild-type allele and the 6373 bp amplicon was the *CpRan1*-null mutant allele that replaced the *CpRan1* gene. The *CpRan1*-null mutant allele was substituted with the replacement vector lacking the genomic region between 13 bp and 1047 bp of the *CpRan1* gene relative to the start codon. Thus, we concluded that the putative *CpRan1*-null mutant appeared to be a heterokaryon, consisting of two genetically different nuclei in a common cytoplasm: one for the wild-type and the other for the *CpRan1*-null mutant allele.

### 3.4. Heterokaryon Analysis of the Putative CpRan1-Null Mutant

**Figure 3:**
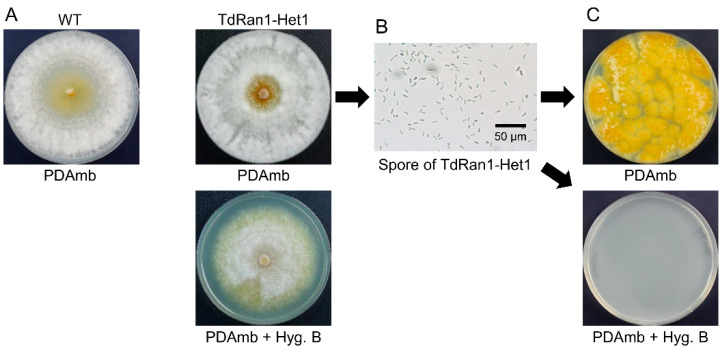
Identification of the *CpRan1*-null mutant and segregation of conidia of the *CpRan1*-null mutant strain. (**A**) The colony morphology after 10 days of culturing is shown. A further extension of the incubation period did not lead to sporulation on PDAmb with hygromycin B. No mycelial growth of conidia was observed on selective PDAmb media even when the incubation period was extended. (**B**) Conidia harvested from the putative *CpRan1*-null mutant strain grown on PDAmb for more than 14 days are shown. (**C**) 100 conidia were spread on PDAmb plates with or without hygromycin B. Strain identifications are provided above the picture. WT and TdRan1-Het1 denote the EP155/2 and heterokaryotic *CpRan1*-null mutant strains, respectively.

Since an asexual spore (conidium) of *C. parasitica* is an aseptate monokaryon, i.e., a single cell with a single nucleus [32], single-spore isolation is the common biological method we used to obtain progeny that have resolved the heterokaryotic state of the putative *CpRan1*-null mutant. Conidia were harvested from putative *CpRan1*-null mutant colonies, microscopically inspected (Figure 3), diluted, and 100 conidia were plated on selective PDAmb medium containing 50 μg/mL hygromycin B. No mycelial growth of conidia was observed on selective PDAmb media even when the incubation period was extended. When the same spore suspension was plated on the non-selective PDAmb media without hygromycin B, 60 to 80 hygromycin sensitive colonies per plate were observed. We performed a genotype analysis using PCR with gene specific primers of the resulting hygromycin sensitive colonies to confirm the genotype of the single-spore progenies from the germinated conidia. PCR analysis of the single-spore progenies revealed the PCR amplicon that corresponds to the wild-type *CpRan1* allele, but not to the *CpRan1*-null mutant allele (Supplementary Figure S4). These results suggest that the conidia suspension consisted of a mixture of two types of conidia: one viable but sensitive to hygromycin B and containing the wild-type *CpRan1* allele, and the other non-viable and most likely containing the *CpRan1*-null mutant allele. These results clearly suggested that the conidia suspension consisted of a mixture of two types of conidia: one was viable but hygromycin B-sensitive containing the wild-type *CpRan1* allele and another that was non-viable most likely containing the *CpRan1*-null mutant allele.

Complementation of the putative *CpRan1*-null mutant strain was conducted using protoplasts from the parental heterokaryotic strain and the complementing vector consisting of the wild-type allele of *CpRan1* and the geneticin-resistance selection marker. Complemented strains showing stable geneticin resistance were selected. Conidia were harvested from the selected geneticin-resistant complemented strains and plated on the non-selective media as well as selective media containing hygromycin B and/or geneticin. Colonies displaying both hygromycin B and geneticin resistance were selected, and PCR analysis of subsequent progeny revealed the presence of both the wild-type and *CpRan1*-null mutant alleles. Colonies showing resistance to geneticin alone or susceptibility to both hygromycin B and geneticin revealed the presence of only the wild-type *CpRan1* allele.

These results suggested that an absence of the essential *CpRan1* gene is lethal. We confirmed that the parental mutant strain was a heterokaryon consisting of two different types of nuclei, i.e., one for the wild-type and the other for the *CpRan1*-null mutant allele.

### 3.5. Characteristics of Heterokaryons

During comparison of cultural characteristics of the parental heterokaryon (TdRan1-Het1) with the wild-type EP155/2, there were no differences observed between TdRan-Het1 and the wild type on a non-selective medium (Figure 3A).

**Figure 4:**
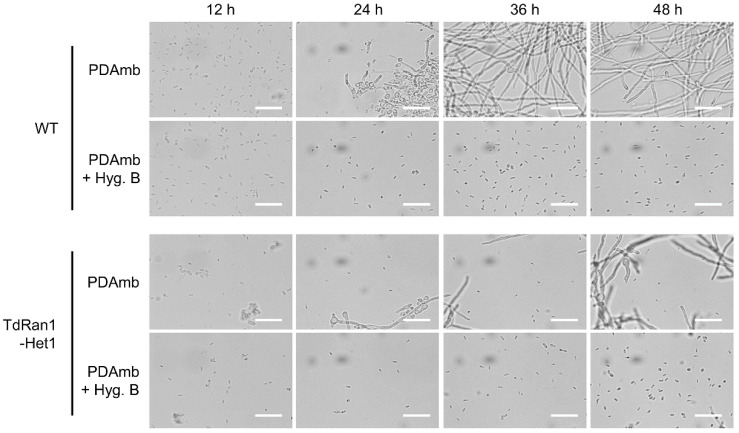
Microscopic observation of germinating conidia. Conidia are observed at every 12 h until 48 h, and the incubation time is shown above the panel. The strains compared are wild-type EP155/2 (WT) and heterokaryotic *CpRan1*-null mutant (TdRan1-Het1). The culture media indicated on the left of the panel are potato dextrose broth (PDB) and hygromycin B-supplemented PDB (PDB + Hyg. B). Scale bar = 50 μm.

In order to analyze the terminal phenotype of the mutant allele, we microscopically observed the conidial germination (Figure 4). Although a large proportion of conidia germinated and resulted in actively growing young hyphae, there were a large number of residual conidia with the mutant allele showing no signs of germination such as swelling or germ tube formation. These results indicated that *CpRan1* is required from the beginning of fungal cell metabolism and suggested that the nucleocytoplasmic transport by Ran protein is fundamental to affect numerous cellular process.

**Figure 5:**
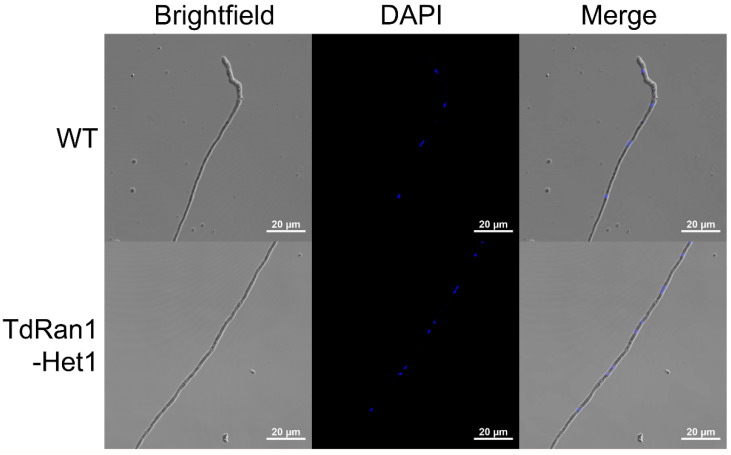
DAPI-stained nuclei of wild-type and the *CpRan1*-null mutant mycelia. Brightfield: Light microscopic images of mycelia. DAPI: DAPI-stained images of corresponding brightfield mycelia. Merge: Merged images are shown. Strains are indicated on the left. WT and TdRan1-Het1 indicate wild-type EP155/2 and heterokaryotic *CpRan1*-null mutant strain, respectively.

Once we have confirmed the existence of the *CpRan1*-null mutant nuclei, we have tested for any discernable changes in the nuclei of heterokaryon using DAPI staining. As shown in Figure 5, the wild type demonstrated the expected well-spaced single nuclei within a single cell demarcated by septa, while the heterokaryon tended to have more than one nucleus per cell. However, we observed no difference in the intensity and foci between the DAPI-stained nuclei in the heterokaryon and the wild type, suggesting that the protein product of the *CpRan1* gene from the wild-type nuclei was able to in trans complement the absence of the Ran protein of mutant nuclei. These results suggest that the *CpRan1* gene is critical from the earliest stages of hyphal growth and that the absence of the *CpRan1* gene, and the consequential absence of a nucleocytoplasmic transport system, affects all of the Ran protein’s functions, including nuclear transportation, nuclear assembly, mRNA processing, and cell cycle control [21,22,23,24,25].

**Figure 6:**
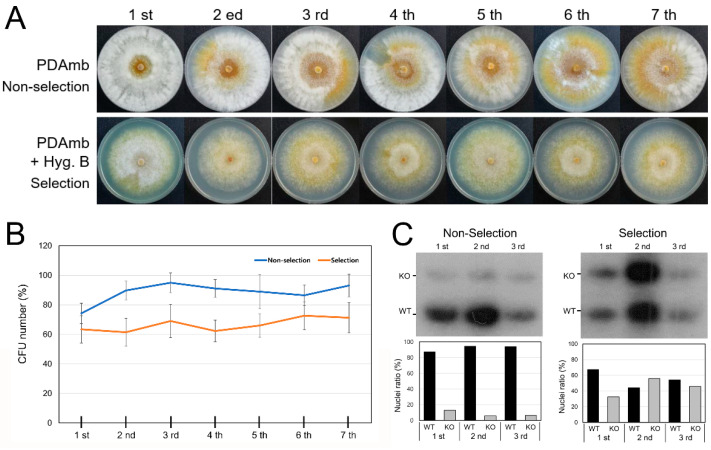
Colony morphology of heterokaryon with and without continuous hygromycin B selection pressure with continuous passage. (**A**) Morphology of *CpRan1*-null mutant (TdRan1-Het1) on nonselective, PDAmb, (upper panel) and selective, PDAmb + Hyg. B; PDAmb supplemented with hygromycin B, (lower panel) media. The number of passages was displayed on the top of the plate. (**B**) Number of germinating spores (CFU) harvested from TdRan1-Het1. (**C**) Southern blot analysis of wild-type (WT) and *CpRan1*-null (KO) nuclei after the successive cultures on non-selective and selective media (upper panels). Densitometry of the hybridizing bands in the corresponding upper panel using Image J. PDAmb and PDAmb + Hyg. B are used for the non-selective and selective media, respectively.

Since DAPI staining suggests the maintenance of the integrity of the nuclei of both wild-type as well as mutant strains and the genotype frequency in a heterokaryon can vary depending culture conditions, we examined the forced changes in the genotype frequency of heterokaryon progeny. As shown in Figure 6, we cultured the heterokaryon strain on selective medium and passaged it every fifth day, forcing the heterokaryon to maintain the high ratio of the *CpRan1*-null mutant nuclei. Initially, 30% of plated conidia did not germinate, i.e., were lethal, and a ratio of no less than 40% remained thereafter. When the heterokaryon was successively cultured on non-selective medium, the ratio of CFU gradually increased to 90% and remained. Thus, depending on the culture conditions, the ratio of viable to nonviable conidia most likely representing wild-type nuclei to *CpRan1*-null mutant nuclei could be varied, ranging from 60 to 90%. However, depending on the genotype frequency, no significant changes in the colony morphology of the heterokaryon were observed.

**Figure 7:**
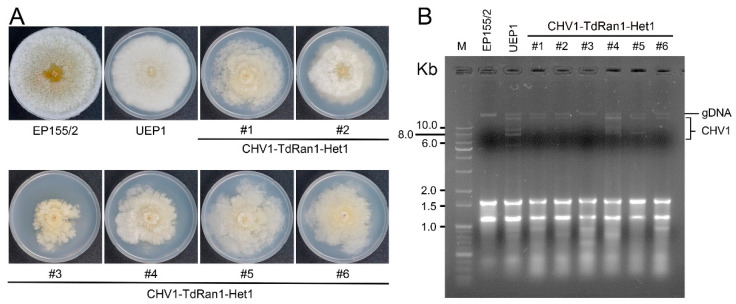
Effect of CHV1 infection to heterokaryons. (**A**) Colony morphology of CHV1-infected heterokaryotic *CpRan1*-null mutant. Strains included are virus-free (EP155/2), a virus-infected isogenic strain (UEP1), and six representative CHV1-infected heterokaryotic progenies (CHV1-TdRan1-Het1 #1-#6). Strains are marked at the bottom of the plate. (**B**) Gel electrophoresis of dsRNA isolated from 0.1 g of lyophilized mycelia.

Hypovirus CHV1 transfection via hyphal fusion was performed by co-culturing the CHV1-infected hypovirulent UEP1 strain and the heterokaryon. Changes in the colony morphology of the CHV1-recipient heterokaryon were observed during the co-culture. Mycelia showing a different morphology were transferred on a selective medium. After the successive transfer of hyphal tips on a selective medium, the presence of CHV1 in the resulting heterokaryon was confirmed by dsRNA preparation followed by agarose gel electrophoresis. Compared to the CHV1-infected UEP1 strain, the CHV1-infected heterokaryon displayed similar viral symptoms such as reduced pigmentation and conidiation (Figure 7). No changes in CHV1 titer were observed in the CHV1-infected heterokaryon.

### 3.6. Functional Analysis of the Essential CpRan1 Gene Using Heterokaryons

**Figure 8:**
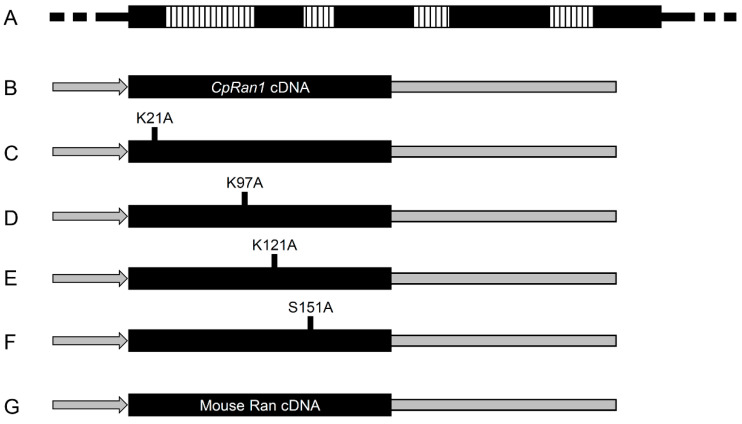
Schematic representation of the complementing vectors. (**A**) Full-length genomic DNA of the *CpRan1* gene was used as a control; vertical dashed lines in the genomic DNA represent introns. (**B**–**F**) A series of vector constructs containing various point mutations of *CpRan1* were constructed using cDNA of the *CpRan1* gene and an expression cassette consisting of a strong constitutive cryparin promoter (p188) and *trpC* terminator. (**G**) A cDNA clone of *Ran* gene from mouse (GenBank No. AAH83356.1) was used for the heterologous expression.

Heterokaryosis is an efficient biological feature to rescue lethal or recessive genotypic nuclei with the help of wild-type nuclei. In this way, various chimeric structures of the corresponding essential gene are transferred to the mutant nuclei. With nuclei segregation using single-spore isolation, it is possible to analyze whether the specific domains or residues of the protein product of an essential gene are necessary for the associated biological function. In this study, we made use of the *CpRan1* heterokaryon (TdRan1-Het1) to analyze the biological function of important amino acid residues. We conducted in silico analysis to select amino acid residues, which are conserved and suggested as being involved in representative post-translational modifications. Four amino acid residues thought to be involved in succinylation (K21), ubiquitination (K97), ubiquitination (K121), and phosphorylation (S151), were selected and analyzed for their importance to the protein product of the *CpRan1* gene. Various complementing vectors with a point mutation of a target amino acid to alanine were used to transform TdRan1-Het1. The resulting complemented transformants were analyzed for the presence of transformed conidia with resistance to both selection markers of hygromycin B as well as geneticin (Figure 8). For each construct, at least 60 geneticin-resistant transformants were selected and cultured on PDAmb and PDAmb supplemented with hygromycin B or geneticin (Table 1). Then, transformants showing resistance to both hygromycin B and geneticin were further cultured for harvesting spores, and the resulting spores were plated on PDAmb and PDAmb supplemented with hygromycin B or geneticin (Supplementary Figure S5A). Except for complementation with genomic DNA, PCR amplification of the various transforming *CpRan1* genes, which use primers from introns, was applied to rule out the possible contamination of heterokaryotic mycelial fragments (Supplementary Figure S5B). In addition, PCR amplification of the transforming gene followed by sequencing was conducted to verify the corresponding gene. Among tested specimens, genomic as well as cDNA clones of the *CpRan1* gene were able to complement the *CpRan1*-null mutant gene to result in canonical colony morphology. The resulting complemented strains showed a similar phenotype as the wild-type EP155/2. Among those tested with the chimera of the *CpRan1* gene, K97A is the only one to complement the *CpRan1*-null nuclei, i.e., conidia growing on both hygromycin B- and geneticin-supplemented media were obtained. Thus, we concluded that K97 residue was not necessary for the function of the *CpRan1* gene and that the K97A mutation was able to support the fungal growth. However, changes in K21, K121, and S151 residues resulted in a lethal phenotype suggesting that these residues are necessary for the function of the *CpRan1* gene. Additionally, transformants complemented by K97A showed the characteristic colony morphology of the wild-type EP155/2, indicating that K97 residue is not essential and does not play any role in the full function of the protein product of the *CpRan1* gene. Interestingly, the gene from *M. musculus* was able to complement the mutant nuclei to grow a colony, but the complemented strain showed a slightly different colony morphology from the wild type in which delayed pigmentation was observed.

## 4. Discussion

Ran is an essential component of the nucleocytoplasmic transport and is implicated in diverse cellular metabolic processes [21,22,23,24,25]. In silico analysis of the genome database of *C. parasitica* demonstrates that there is only one Ran gene, *CpRan1*, in the genome of *C. parasitica*. Based on the discrepancy between proteomic and transcription analysis, it appears that the regulation of the expression of the *CpRan1* gene is multilayered and numerous factors can affect *CpRan1* expression.

It is not uncommon for proteomic analysis to show different expression patterns from transcriptomic analysis [20], especially when applied to genes regulated at post-transcriptional, translational, and post-translational levels [41,42]. In our expression analysis results that used cultures under the same conditions, i.e., 24 h after the transfer to the TA-supplemented media, the qRT-PCR analysis showed a slight but significant increase in the *CpRan1* transcripts, while the corresponding protein spot displayed a larger two-fold decrease [20]. Moreover, dramatic changes in the accumulation of *CpRan1* transcripts were observed by the CHV1 infection under the same culture conditions. Northern blot analysis at 24 h after the transfer to the TA-supplemented media was consistent with the qRT-PCR analysis, suggesting that the increase in the amount of *CpRan1* transcripts was not mere measurement noise. The differences between mRNA and protein expression results suggested a regulatory process after the transcriptional level. Such regulatory processes are central to explain the abundance of the protein product of the *CpRan1* gene, which is critically modulated by the CHV1 infection and TA supplementation. Additionally, upregulation of the *CpRan1* transcripts by the CHV1 infection were greater on solid medium than in liquid culture conditions, confirming that *CpRan1* gene expression is also seriously affected by different culture conditions. In sum, our results indicate that the regulation of *CpRan1* gene expression is complex and implicates numerous factors at various levels.

The high ratio of the wild-type nuclei over the *CpRan1*-null mutant nuclei was interesting. The high ratio of the wild-type nuclei of the *CpRan1* heterokaryon (TdRan1-Het1) was maintained on the non-selective media (90%) and even after the successive cultures on selective media (>60%). This high ratio wild-type *CpRan1* nuclei was 40% to 70% greater than those of other essential genes such as *CpCdc48* and *CpRbp1* [28,31]. This is indicative of how *C. parasitica* requires a large amount of protein product from the *CpRan1* gene to fulfill its fundamental metabolic purpose, which includes nucleocytoplasmic transport. This may simply imply how much gene product is required to maintain the heterokaryon, which indicates that many genes are essential, but the amount of each gene product varies to maintain normal cell metabolism.

A heterokaryon is defined by the presence of two or more genetically different nuclei in a common cytoplasm. It is a unique genetic characteristic of fungi, which allows it to maintain and proliferate the nuclei of recessive genotype with the help of proficient wild-type nuclei. Utilizing the *CpRan1* heterokaryon, we were able to proliferate *CpRan1*-null mutant nuclei and complement in trans the *CpRan1*-null mutant nuclei with various chimeric constructs of the *CpRan1* gene. Our complementation analysis using the heterokaryon and various chimeric gene constructs is a proof-of-concept experiment that validates the feasibility of functional analysis of amino acid residues within the protein product of an essential gene. This differs from our previous study in which we illustrated that heterokaryons can be used to determine the necessity of specific protein domains through a domain swapping approach and did not examine the role of specific amino acid residues [28]. Among the four post-translationally modified amino acid residues suggested in silico, our complementation verified that three residues of K21, K121, and S151 played an important role in the biological functioning of the Ran protein. While further studies on the nature of modification will be required to confirm and refine this method, our results suggest that the post-translational modification of *CpRan1*, as predicted in silico, does exist and is important to biological functioning. It is possible that the failure of complementation using various chimeric constructs is the consequence of using a heterologous strong constitutive, instead of a native, promoter to express the *CpRan1* gene. However, the same strong constitutive promoter was able to complement the *CpRan1*-null mutant to drive full-length cDNA expression. The strong constitutive promoter was also used for the successful expression of the heterologous Ran gene from *M. musculus*. Ultimately, therefore, we do not attribute the failure to complement with the mutated amino acid residues to the changes in the expression level of the mutated *CpRan1* gene by the strong constitutive promoter, but rather to true changes in the functionality of the altered amino acid residues. In contrast, the K97A mutation did not affect the ability of Ran1 to complement the knock out, suggesting either that the ubiquitination of K97 predicted in silico did not occur or, if it did, that the post-translational ubiquitination was no longer necessary to biological functioning. In addition, the success of our complementation, which used a heterologous gene such as the Ran gene from *M. musculus*, is very promising. The Ran protein is evolutionarily conserved to the point that the amino acid sequence of the Ran protein from *M. musculus* (GenBank No. AAH83356.1) is similar to other mammalian homologues and is identical to the human Ran gene (GenBank No. AAH16654.1). Our heterokaryon therefore has utility in the functional analysis of heterologous genes from other organisms in which it does not exist. Our recent study on the Ran-binding protein gene *CpRbp1*, the modulator of Ran, demonstrated that the *CpRbp1* gene is essential, which is consistent with the current studies showing the necessity of the *CpRan1* gene. In *C. parasitica*, four essential genes including the *CpRan1* gene have been verified by heterokaryon analysis indicating a well-balanced maintenance of genetically engineered-lethal nuclei [28,30,31]. This might represent the increased tendency of heterokaryon formation in essential genes in *C. parasitica,* even though the stable heterokaryon has been reported in nature [29]. Considering heterologous complementation, our heterokaryon analysis using *C. parasitica* can be further extended to examine the essential genes of other organisms. Our analysis aids the straightforward detection of essential genes, convenient resolution by single sporulation, and balanced proliferation of genetically recessive nuclei.

In our previous studies, we mimicked the host environment by supplementing tannic acid and revealed several genes that were regulated by tannic acid and/or CHV1 [20]. Among them, three genes, *CpCdc48, CpRbp1*, and *CpRan1*, were verified to be essential [28,31]. Further studies will be required to analyze why these essential genes are affected under the conditions of TA supplementation, which is also known as the host defense barrier. It will also be important to understand whether in general it will be necessary to have these essential genes modulated for pathogenesis in other fungi.

In this study, we characterized the putative *CpRan1*-null mutant to be a heterokaryon consisting of two different types of nuclei carrying either the wild-type or the *CpRan1*-null mutant allele in its common cytoplasm. Heterokaryon analysis indicated that the Ran gene of the chestnut blight fungus, *C. parasitica*, is essential, and the loss-of-function is lethal. Microscopic observation of conidia containing mutant nuclei suggested that the *CpRan1* gene plays important roles in early germination and is necessary for fundamental cellular function. We demonstrated a successful functional analysis of the *CpRan1* gene by complementation of heterokaryons using various chimeric constructs of the *CpRan1* gene. Heterokaryon analysis of the essential gene allow us to increase the likelihood of applying our knowledge of this fungus to the analysis of the structure–function relationship of a conserved essential gene.

## Figures and Tables

**Table 1:** Numbers of transformants using various complementing *CpRan1* vectors on different selection media and numbers of resulting hygromycin B-resistant transformants used for spore harvesting and single-spore analysis.

Vector Constructs in Figure 8	Number of Transformant		CFU		
	PDAmb + Geneticin	PDAmb + Hyg. B	PDAmb	PDAmb + Geneticin	PDAmb + Hyg. B
gDNA	280	154	23	23	17
cDNA	68	8	8	8	6
K21A	60	0	-	-	-
K97A	74	7	7	7	5
K121A	65	0	-	-	-
S151A	60	0	-	-	-
Mouse	80	9	9	9	5

## Supplementary Materials

The following are available online at https://www.mdpi.com/article/10.3390/jof7050332/s1, Figure S1: Characterization of *CpRan1* gene, Figure S2: Northern blot analysis of *CpRan1* in response to hypovirus infection and TA supplementation, Figure S3: PCR amplicons of the heterokaryotic *CpRan1*-null mutant, Figure S4: PCR amplicons of the hygromycin sensitive colonies from the germinated conidia, Figure S5: Complementing analysis of various chimeric structures of *CpRan1* gene.
