# Supplementary material for: Functional Analysis of an Essential GSP1/Ran Ortholog Gene, CpRan1, from the Chestnut Blight Fungus Cryphonectria parasitica Using a Heterokaryon
Source: J Fungi (Basel). 2021 Apr 25;7(5):332. doi: 10.3390/jof7050332 (PMC8146580; doi:10.3390/jof7050332)
Supplement: Supplementary file 1 [file jof-07-00332-s001.zip › jof-1161484-supplementary.pdf]

Functional analysis of an essential GSP1/Ran ortholog gene, *CpRan1*, from the chestnut blight fungus *Cryphonectria parasitica* using a heterokaryon

Yo-Han Ko, Jeesun Chun, and Dae-Hyuk Kim*

Department of Molecular Biology, Institute for Molecular Biology and Genetics, Jeonbuk National University, 567 Baekje-daero, Jeonju, Chonbuk 54896, Korea

* Corresponding authors. Address: Department of Molecular Biology, Department of Bioactive Material Sciences, Jeonbuk National University, Jeonju, Chonbuk 54986, Korea. Tel: +82 63 270 3440, Fax: +82 63 270 4312.

E-mail address: dhkim@jbnu.ac.kr (D.H. Kim).

Keywords: *Cryphonectria parasitica*, Ran, Heterokaryon, Hypovirulence

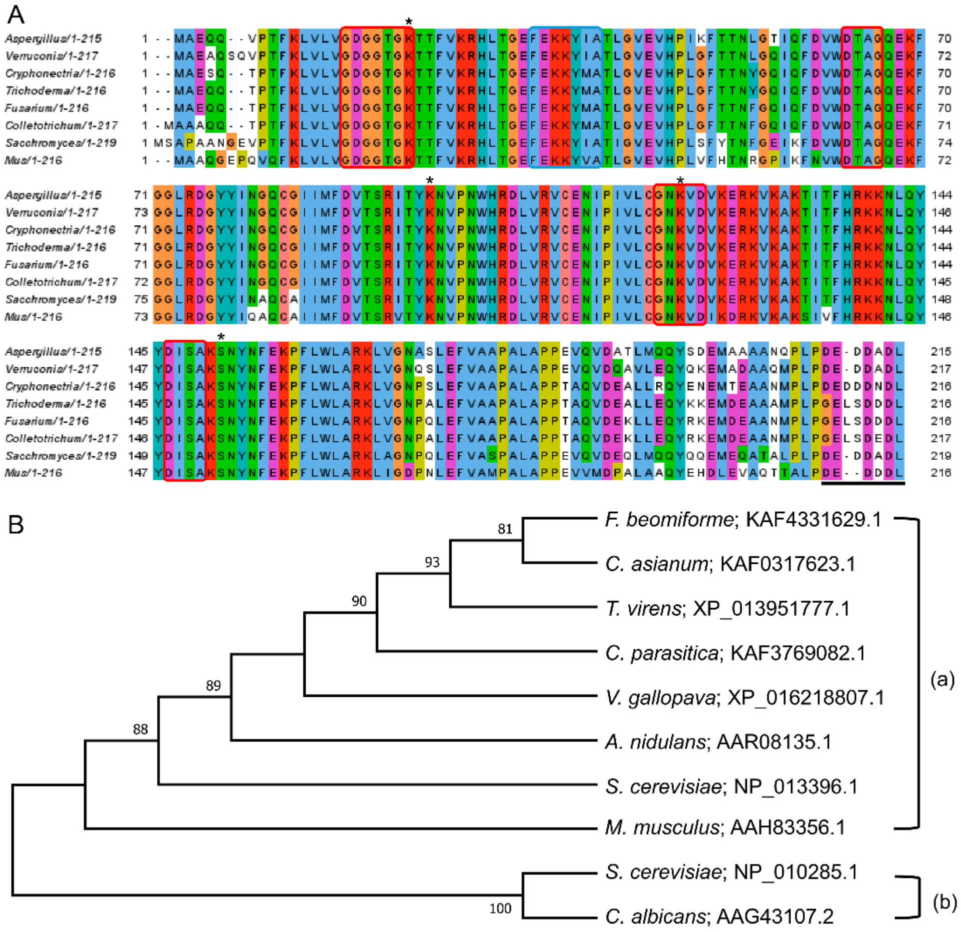

Supplementary Figure S1. Characterization of *CpRan1* gene. (**A**) Alignment of GSP1/Ran proteins. Amino acid sequence of GSP1/Ran protein is highly conserved. Four guanine nucleotide-binding domains, an effector domain, and an acidic C-terminal domain are represented by red boxes, blue box, and underline, respectively. Asterisks represent *in silico*–suggested post-translational modification sites, which have been modified for the functional analysis. (**B**) Phylogenetic tree based on Maximum-likelihood algorithm using JTT matrix [1] constructed using MEGA7 [2] from a comparative analysis of GSP1/Ran proteins (a) and Ran binding proteins as an outgroup (b). Numbers at nodes are levels of repeatability for branch points on 1,000 bootstraps, and values are shown only if greater than 70%.

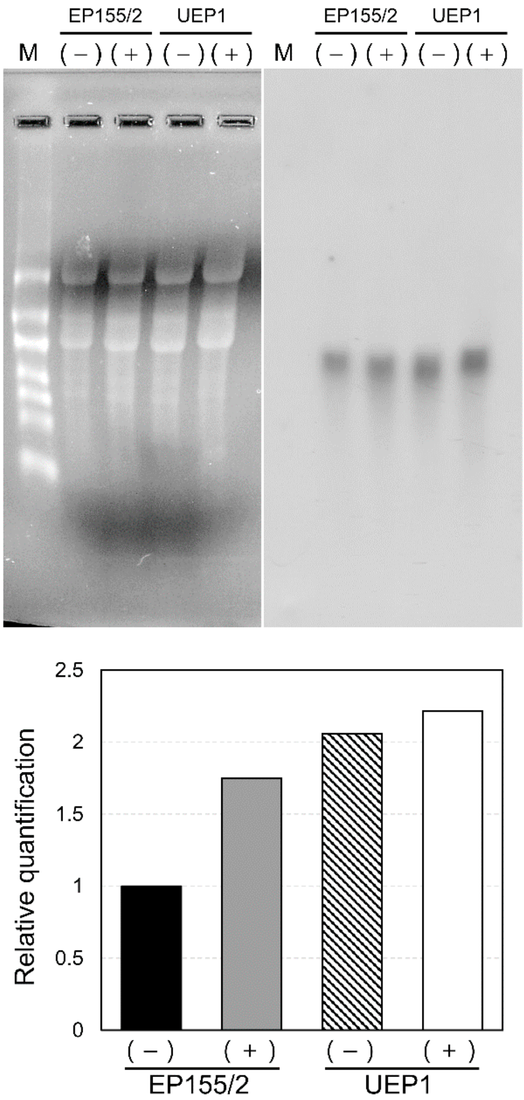

Supplementary Figure S2. Northern blot analysis of *CpRan1* in response to hypovirus infection and TA supplementation. Total RNA was extracted from EP155/2 and UEP1 24 h after the transfer. Identification of the strains is shown at the top of the lanes; (+) and (–) above the panel indicate with and without TA supplementation, respectively. Equal loading of RNA samples is shown in the left panel by the ethidium bromide-stained gel (rRNA). Densitometry of the hybridizing bands in the corresponding upper panel using Image J software is shown below.

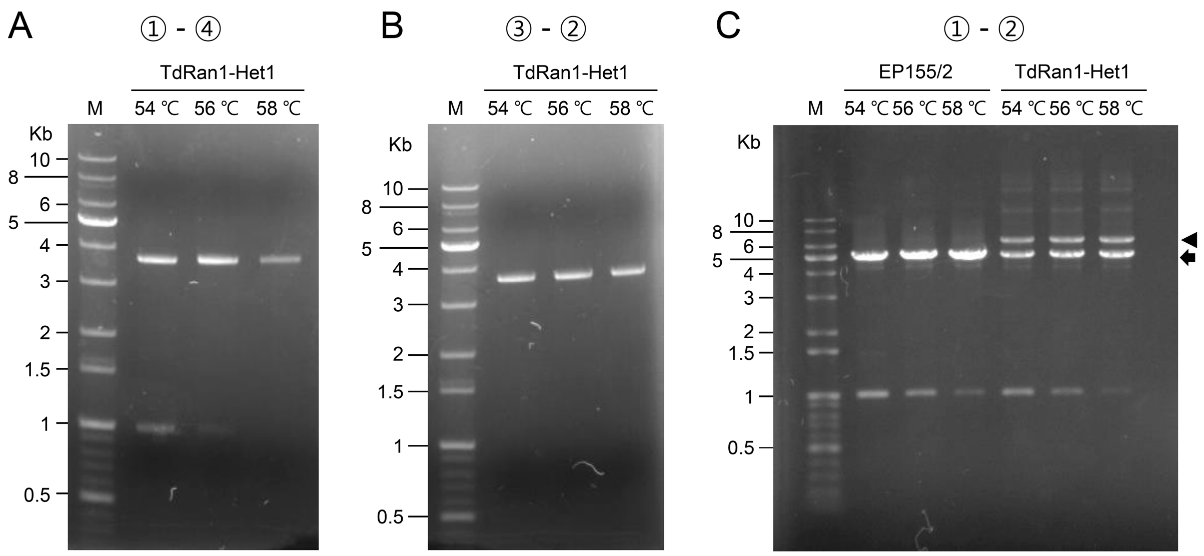

Supplementary Figure S3. PCR amplicons of the heterokaryotic *CpRan1*-null mutant. Ethidium bromide-stained gel of PCR amplicons of the heterokaryotic *CpRan1*-null mutants (TNFs) and the wild type EP155/2 strain using the primer pairs 1 & 4 (A), 2 & 3 (B), and 1 & 2 (C) in Fig 2A. Lane M contains the 1.0 kb size marker. Primers used for strain confirmation are indicated by arrows in the restriction map in the Fig 2A. PCR amplicons resulting from the use of the primer pair 1 & 2 marked by arrow and arrowhead indicate the wild type and the null-mutant alleles of *CpRan1*, respectively. Note that PCR amplicons using gradient PCR with different annealing temperatures (54 °C, 56 °C, and 58 °C) are depicted, and that the non-specific small-sized bands associated with primer pair 1 become less conspicuous when higher temperatures are used (A and C).

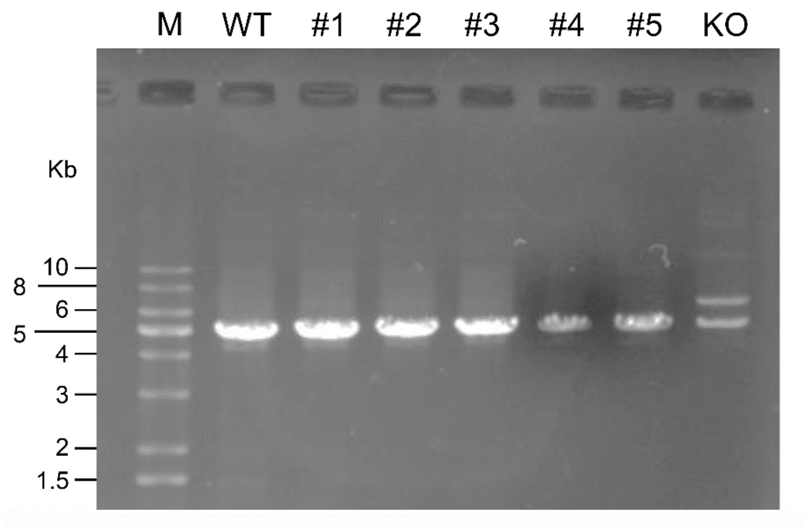

Supplementary Figure S4. PCR amplicons of the hygromycin sensitive colonies from the germinated conidia. Ethidium bromide-stained gel of PCR amplicons of the wild type EP155/2 strain (WT), five randomly selected hygromycin sensitive colonies (#1 - #5), and the heterokaryotic *CpRan1*-null mutant (KO) using the primer pair 1 & 2 in Fig 2A. Lane M contains the 1.0 kb size marker. Primers used for strain confirmation are indicated by arrows in the restriction map in the Fig 2A. Note that PCR analysis of the hygromycin sensitive colonies exhibits the PCR amplicon that corresponds to the wild type *CpRan1* allele, but not to the *CpRan1*-null mutant allele.

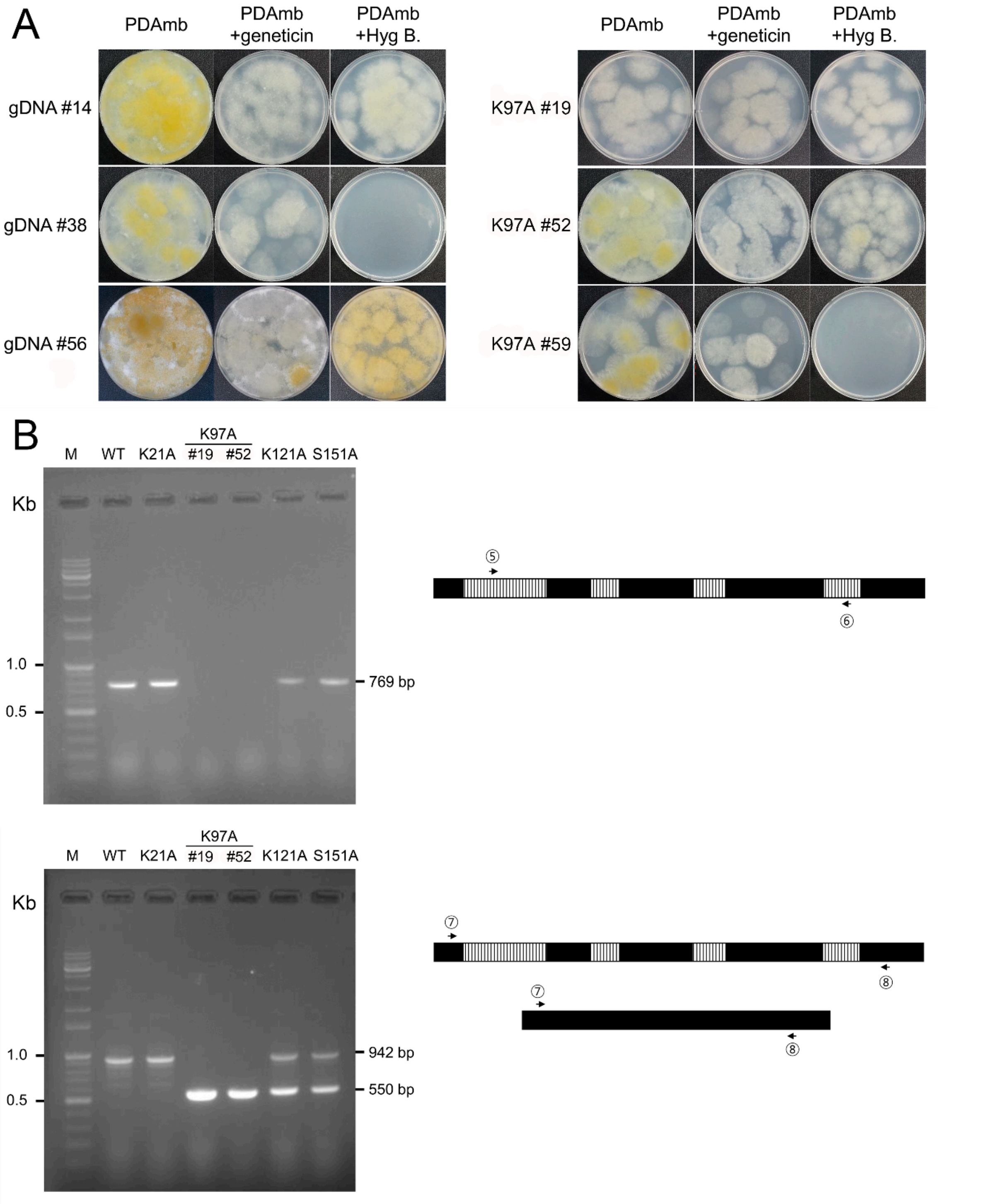

Supplementary Figure S5. Complementing analysis of various chimeric structures of *CpRan1* gene. (**A**) Conidia of complemented strain were spread on PDAmb and PDAmb supplemented geneticin or hygromycin B. Strains are indicated on the left of plates. Note that a large number of CFUs on PDAmb + Hyg. B indicate that the complementing structure is functional while no CFU on PDAmb + Hyg. B indicates that the

complementing structure is not functional. (**B**) Ethidium bromide-stained agarose gel of PCR amplicons of complemented strains using the primer pair 5 & 6 (upper panel), and 7 & 8 (bottom panel). Note that the absence of PCR amplicon with primers 5&6 but the presence of an expected-size PCR amplicon with primers 7 & 8 indicate that single-spored progenies of complemented transformants are pure complemented strain not heterokaryon nor mixed cultures. Lane M contains the 1.0 kb size marker. In *CpRan1* gene, exon is solid and intron is vertical mark.

Supplementary Table S1. List of PCR primer sequences.

Primer	Name	Primer Sequence (5′-3′)	Use
1	CpRan1-gF1	GGCAAAGGACGAGAGCTGTGTG	
2	CpRan1-gR1	TGCCTGGTCCTAATCCCCAAGA	
3	Hph-F2	ATTTCATATGCGCGATTGCT	
4	Hph-R1	ACCCGAAAACGCGTTTTATT	
5	Ran_intron_F	GCCGAGCTACCTCCCTCT	
6	Ran_intron_R	CAGCAAACATGACGCGCA	
7	Ran_exon-F	GCTCGTCCTTGTTGGCGA	
8	Ran_exon-R	GCGCAGAAGGGCCTCATC	
9	RT-Gpd-F	CCGTCAACGACCCCTTCAT	
10	RT-Gpd-R	GTTGCCGTGTTGAGAGTCATAC	
11	RT-Ran1-F1	TGTTACCTCCCGTATTACCTACAAGA	
12	RT-Ran1-R1	GTTCTCGCAGACACGAACGA	
13	CpRan1-cF1	CAACAACGATCATGGCCG	
14	CpRan1-cR1	CGCATTTCGCCCTCTACA	
15	Ran 5′-F1	GATATCATCGACCTCGCCTCA	
16	Ran 5′-R1	TCCTTCAATATCATCTTCTGTCGACG ACTCTCGGCCATGATCGTT	Construction of CpRan1-null mutant. Underline indicated the hygromycin B resistant cassette (Hyg. B) part.
17	Ran-Hph-F1	AACGATCATGGCCGAGAGTCGTCGA CAGAAGATGATATTGAAGGA	Construction of CpRan1-null mutant. Underline indicated the hygromycin B resistant cassette (Hyg. B) part.
18	Hph-Ran-R2	TCCCGAGCATCGCATTTCGGTCGAC GCTCTCCCTTATGC	Construction of CpRan1-null mutant. Underline indicated the hygromycin B resistant cassette (Hyg. B) part.
19	Ran 3′-F2	GCATAAGGGAGAGCGTCGACCGAA ATGCGATGCTCGGGA	Construction of CpRan1-null mutant. Underline indicated the hygromycin B resistant cassette (Hyg. B) part.
20	Ran 3′-R2	TGCCTTGGGATTCAGATCGT	

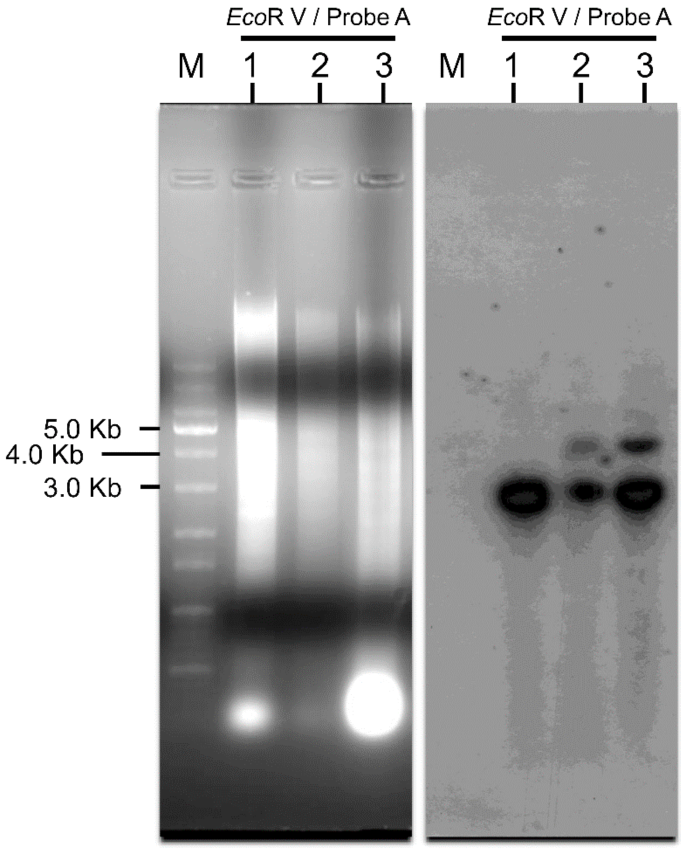

Full-length gel and blot of Figure 2

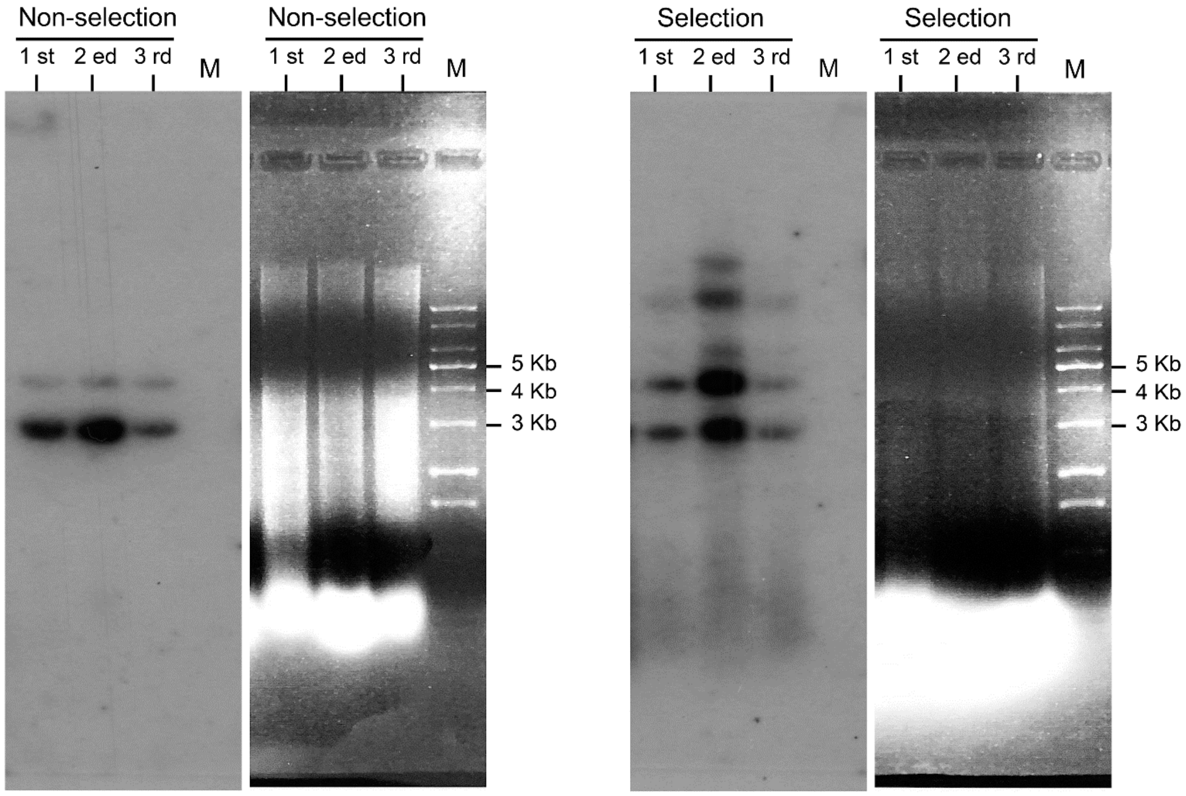

Full-length gel and blot of Figure 4

References

1. Jones, D.T.; Taylor, W.R.; Thornton, J.M. The rapid generation of mutation data matrices from protein sequences. *Comput. Appl. Biosci.* **1992,** *8,* 275-282.
2. Kumar, S.; Stecher, G.; Tamura, K. MEGA7: Molecular ecvolutionary genetics analysis version 7.0 for bigger datasets. *Mol. Biol. Evol.* **2016,** *33,* 1870-1874.
